# Metabolomics Identifies Multiple Candidate Biomarkers to Diagnose and Stage Human African Trypanosomiasis

**DOI:** 10.1371/journal.pntd.0005140

**Published:** 2016-12-12

**Authors:** Isabel M. Vincent, Rónán Daly, Bertrand Courtioux, Amy M. Cattanach, Sylvain Biéler, Joseph M. Ndung’u, Sylvie Bisser, Michael P. Barrett

**Affiliations:** 1 Wellcome Trust Centre of Molecular Parasitology, Institute of Infection, Immunity and Inflammation, College of Medical, Veterinary and Life Sciences, University of Glasgow, Glasgow, United Kingdom; 2 Glasgow Polyomics, College of Medical, Veterinary and Life Sciences, University of Glasgow, Glasgow, United Kingdom; 3 INSERM U1094, Tropical Neuroepidemiology, Limoges, France; Université de Limoges, Institute of Neuroepidemiology and Tropical Neurology, Limoges, France; 4 Foundation for Innovative New Diagnostics, Geneva, Switzerland; International Centre of Insect Physiology and Ecology, KENYA

## Abstract

Treatment for human African trypanosomiasis is dependent on the species of trypanosome causing the disease and the stage of the disease (stage 1 defined by parasites being present in blood and lymphatics whilst for stage 2, parasites are found beyond the blood-brain barrier in the cerebrospinal fluid (CSF)). Currently, staging relies upon detecting the very low number of parasites or elevated white blood cell numbers in CSF. Improved staging is desirable, as is the elimination of the need for lumbar puncture. Here we use metabolomics to probe samples of CSF, plasma and urine from 40 Angolan patients infected with *Trypanosoma brucei gambiense*, at different disease stages. Urine samples provided no robust markers indicative of infection or stage of infection due to inherent variability in urine concentrations. Biomarkers in CSF were able to distinguish patients at stage 1 or advanced stage 2 with absolute specificity. Eleven metabolites clearly distinguished the stage in most patients and two of these (neopterin and 5-hydroxytryptophan) showed 100% specificity and sensitivity between our stage 1 and advanced stage 2 samples. Neopterin is an inflammatory biomarker previously shown in CSF of stage 2 but not stage 1 patients. 5-hydroxytryptophan is an important metabolite in the serotonin synthetic pathway, the key pathway in determining somnolence, thus offering a possible link to the eponymous symptoms of “sleeping sickness”. Plasma also yielded several biomarkers clearly indicative of the presence (87% sensitivity and 95% specificity) and stage of disease (92% sensitivity and 81% specificity). A logistic regression model including these metabolites showed clear separation of patients being either at stage 1 or advanced stage 2 or indeed diseased (both stages) versus control.

## Author Summary

Human African trypanosomiasis, also known as sleeping sickness, is a parasitic disease that affects people in sub-Saharan Africa. There are two stages of the infection. The first stage involves parasites proliferating in the bloodstream following introduction via the bite of an infected tsetse fly. The second, more serious stage, involves parasite invasion and proliferation within the central nervous system causing characteristic disturbances to the patients’ sleep wake patterns and progressive appearance of other neurological signs, including walking disabilities behaviour changes, abnormal movements, incontinence, then ultimately coma and death. Drugs are available to treat both stages of the disease, but the drugs for stage 2 disease have serious side effects and must be administered in hospital settings. Stage determination is thus a key element for disease management. Currently staging involves microscopic evaluation of CSF following a lumbar puncture. Here, we have analysed the metabolome of CSF, blood and urine of patients to seek biomarkers to stage the disease based on these biofluids. CSF and blood fluids were found to have distinctive metabolic biomarkers and when several of these metabolites are combined, a sensitive and robust discriminatory staging test can be developed. Some CSF metabolic markers relate to brain inflammation, whilst others may be related to somnolence associated with the disease in stage 2 patients, which may also help in understanding disease progression. Interestingly, distinctive biomarkers were also found in plasma, potentially abrogating the need for diagnostic lumbar punctures in the future.

## Introduction

In many fields of medicine, investigators are seeking ways to determine which patients will respond to particular drugs in order to guide therapy. For example, some cancers will respond to particular therapies if they carry faulty alleles of oncogenes whose products are the targets of those drugs. Examples where determination of an individual’s likelihood of response based on precise diagnosis of their disease are currently rare. One condition in which patient stratification has been possible based on available diagnosis is human African trypanosomiasis (HAT). HAT, also known as sleeping sickness, is a parasitic disease of sub-Saharan Africa affecting isolated, rural communities. Two sub-species of the parasite infect humans. *Trypanosoma brucei gambiense* persists in West and Central Africa and is responsible for 90% of cases, while *T*. *b*. *rhodesiense* exists in East and Southern Africa. Uganda is the only country where both sub-species of the parasite exist. Upon infection, through the bite of an infected tsetse fly, parasites multiply in the blood and lymphatic systems of the patient (stage 1), before invading the central nervous system in stage 2 of the disease. Stage 2 disease leads to progressive neurological dysfunction: anxiety, depression, psychotic episodes, disrupted sleep-wake profile, coma and ultimately death if untreated [1][2]. Vaccines to prevent the disease are unlikely to be developed due to a complex process of antigenic variation [3]. Appropriate medication is therefore crucial to control HAT. Patients in stage 1 disease are treated with pentamidine or suramin, but as these drugs do not cross the blood-brain barrier, the highly toxic melarsoprol is required for stage 2 *rhodesiense* disease [4]. Nifurtimox-eflornithine combination therapy is commonly used to treat *gambiense* disease [4]. Two new drugs, fexinidazole and SCYX-7158, that may treat both stages of the disease, are in development [5,6], but these compounds are years away from the clinic and may still fail in clinical trials. Even when they are in use, a staging test will still be required to evaluate efficacy in both stages and as a measure of post-treatment success.

**Fig 1:**
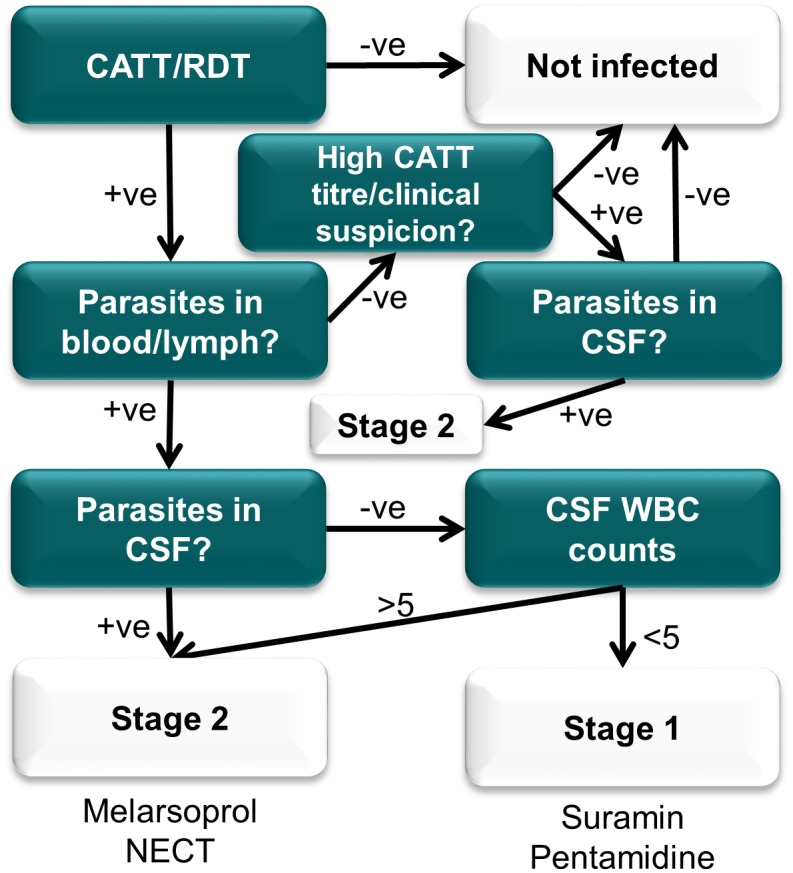
Current diagnosis of HAT. CATT: card agglutination test for trypanosomiasis. CSF: cerebrospinal fluid. RDT: Rapid Diagnostic Test.

Current screening methods for the disease [7] include a serological blood test (for the *gambiense* form of the disease only), followed by blood microscopy then microscopy of cerebrospinal fluid (CSF) to check for parasites. If stage 2 disease is suspected, but no parasites are observed in the CSF, a white blood cell (WBC) count above an arbitrary number (often 5 WBC/μL) may be used for staging (Fig 1). However, the requirement for lumbar puncture, coupled to poor sensitivity, makes staging difficult to perform.

Since the turn of the millennium, the number of cases of HAT have dramatically reduced [8] and in 2014, there were fewer than 4,000 reported cases [9]. HAT was included in the London Declaration of 2012, an agreement between pharmaceutical companies, charities, NGOs and endemic countries that endorsed a roadmap set by the World Health Organisation to build towards elimination of five neglected tropical diseases by 2020 [10]. As a result, there has been a push to develop new drugs, non-pharmaceutical interventions and diagnostic tools for HAT.

As the number of cases of HAT decreases, new ways of diagnosing the disease become more important. Time-consuming microscopy with specialised technicians is no longer suitable when large swathes of the population are being screened to search for the few remaining patients. Improved diagnostics are critical for staging HAT and finding patients as the elimination campaign proceeds. Moreover, trials on drugs aimed at bringing therapies to assist in elimination will benefit from tests, preferably not involving lumbar puncture, that are indicative of cure of stage 2.

Although identification of parasites in blood or CSF has remained the gold standard in diagnosing HAT, there has been increasing use of serological markers (circulating antibodies detected by the card agglutination test for trypanosomiasis (CATT) and recently introduced rapid diagnostic tests (RDTs) based on lateral flow devices [11,12] and molecular tools including loop-mediated isothermal amplification (LAMP) of parasite DNA [13]. Elevated IgM and proteins in CSF have also been proposed as possible biomarkers for staging, and a series of CSF and plasma based cytokine markers have all been investigated [14,15]. A CSF related metabolite, neopterin, however, has emerged as the most sensitive molecular biomarker for staging discovered to date [14,15]. A targeted analysis was carried out, given the finding of elevated neopterin in the CSF of patients before, during and after treatment [16]. The value of neopterin as a biomarker was confirmed, and the marker was also found to be predictive of cure following treatment [16]. However, tests based on neopterin still require collection of CSF in order to identify its elevation in infection, and specificity is relatively low given that neopterin is also found in other neurological infections, notably HIV, tuberculosis and malaria, which are frequently co-existent in HAT patients [17–20]. The fact that neopterin is a metabolite, however, emphasises the potential of metabolic biomarkers in diagnostics.

Metabolic biomarkers have been used in the diagnosis of a range of conditions for many years. Diabetes, for example, is diagnosed due to elevated glucose levels in the blood [21], pregnancy tests detect human chorionic gonadotropins [22] and blood creatinine levels [23] are used to identify failures in kidney function. High resolution mass spectrometry can be used to identify a wide range of metabolic species in a technique known as metabolomics [24]. Differences in the abundance of these small (<1200 Da) metabolites in the biofluids of infected individuals may be used to discriminate between different disease states with the aim of developing new diagnostic tools [25] and a recent study made the first tentative investigations into possible biomarkers in HAT patients. This study was limited, however, due to a lack of patient stratification, and was only done in *T*. *b*. *rhodesiense* HAT patients [25].

A simple biomarker-based test for HAT would revolutionise the way that the disease is screened. Microscopes and centrifuges would no longer need to be transported into the field, allowing much greater access to the isolated communities still affected by the parasite. To be successful, the test would need to be stable at a range of temperatures for a long period of time, simple and quick to use without extra equipment, reliable (with a high specificity and low false positive and false negative rates) and cheap. A dipstick format where a colour change indicates the presence of infection would be ideal. These types of tests are possible with metabolic biomarkers linked to a colour change reaction to detect their presence [26].

Here, we report a comprehensive, untargeted metabolomic analysis of human blood, urine and CSF from 16 seropositive (but parasite negative) subjects, 20 stage 1 and 20 advanced stage 2 HAT patients.

## Materials and Methods

Subjects—Samples were obtained retrospectively from a clinical study conducted in Angola between 2008 and 2011. This study aimed to collect appropriate clinical, neurological, psychiatric and biological data from a cohort of 236 *T*. *b*. *gambiense* infected patients followed up prospectively from diagnosis to end of follow-up, and controls. Controls and patients were enrolled during both active and passive screening activities by teams of the national sleeping sickness control program. The reference screening test at that time was the CATT [27] followed by confirmation using microscopy, with concentration methods for blood/CSF. Cases of HAT were defined as subjects in whom trypanosomes were demonstrated either in blood, lymph node aspirate or CSF by microscopy. Cases were classified as stage 1 when no trypanosomes were observed in CSF and when the CSF white blood cell count (WBC) was lower than or equal to 5 WBC/μL, while those with trypanosomes in CSF and/or a CSF WBC count above 20 WBC/μL were classified as advanced stage 2. Controls were subjects living in the same areas as cases, with no previous history of HAT treatment and who were seropositive (positive with one or more serological tests) but with no detectable parasites in any body fluid. All participants were examined clinically and a questionnaire was used to note all clinical and neurological characteristics, including sleep and psychiatric disturbances using the Mini-International neuropsychiatric interview [28] and the Hamilton rating scale for depression. These two scales include sleep examination criteria. All participants were checked clinically and microscopically for the presence of the main parasitic co-infections (malaria by blood smear, filariasis during blood examination by capillary tube centrifugation, and schistosomiasis when blood was detected in urine samples). The HIV and syphilis status was determined retrospectively on stored samples using VIKIA HIV 1/2 test (Biomérieux, France), RPR-Nosticon II (Biomérieux, France) and TPHA test (Biorad, France) respectively. These analyses aimed to exclude subjects with interfering parameters for CSF analysis.

Test procedures and sample collection—Samples were collected in 2008, 2009 and 2010 and were stored in liquid nitrogen before being transported to Limoges on dry ice, then stored at -80°C. Although the significant time lapse between collection and metabolomics analysis could lead to storage related effects on the samples, all samples were treated equally, hence comparisons between samples may be considered robust. The identification of metabolites such as neopterin, behaving as previously demonstrated in targeted analysis [14,15] corroborated this. A lymph node aspirate was taken from any subject who presented with swollen lymph nodes and examined for trypanosomes by microscopy. Ten ml venous blood with heparin as anticoagulant was collected from CATT positive subject, as well as from lymph node positive patients. Six hundred μl of blood was used to perform the capillary tube centrifugation test (4 capillary tubes of 75 μl) and the miniature anion exchange centrifugation technique (300 μl) [29]. For subjects who were positive by CATT on whole blood, 1 ml plasma was used to perform CATT dilutions. Parasitologically confirmed cases and/or subjects found positive by CATT at a dilution of 1/16 who were negative by all other parasitological methods that were performed underwent a lumbar puncture, in accordance with national guidelines for stage determination and/or parasitological confirmation in CSF, when there were suggestive neurological signs. Parasitological examination of CSF was done using the modified single centrifugation technique [29]. This optimised parasitological confirmation method permits sensitivity or parasite detection similar to molecular testing.

All plasma, buffy coat and CSF samples that remained after the diagnostic procedures were aliquoted and stored in liquid nitrogen.

Patient medical data were anonymised.

### Ethics Statement

Ethical clearance was obtained from the “Direccao National de Saude Publica, Ministerio da Saude”. Written informed consent was received from these subjects prior to enrolment and/or from their parents or guardians for participants below 18 years of age. Any individual who declined to participate was followed up according to the standard procedures of the national control programme.

Metabolite extractions—metabolite extractions were performed as per standard procedures [30] in January-March 2015 (after between 5 and 7 years in storage). Samples were checked for metabolite degradation and all passed. Briefly, 5 μL of sample was extracted in 200 μL of UPLC grade chloroform:methanol:water (1:3:1) on ice. Samples were centrifuged and stored at -80°C before being run through the LC-MS system.

LC-MS—Samples were run on a QExactive mass spectrometer (Thermo) after separation on a zic-HILIC column (Sequant) according to previously published methods [30,31]. A 10μL sample injection was used.

Data analysis—Raw data were filtered and aligned using mzMatch [24] then further filtering and putative annotation for metabolic features was conducted using IDEOM [32] version 19 using generous parameters (0.5 minute retention time window for matching to a standard, 3ppm mass error for identification, minimum number of detections of three per group, a peak height intensity filter of 1000 and a relative standard deviation filter of 0.8). Data were exported from IDEOM to MetaboAnalyst [33] PiMP (http://polyomics.mvls.gla.ac.uk/: PCA plots and TICs) and Graphpad Prism (histograms).

Metabolite identification—Metabolic features in this manuscript are named according to their best match based on exact mass, retention time match to an authentic standard, retention time prediction [34], fragmentation pattern match to MzCloud database (https://www.mzcloud.org/home.aspx) and isotope distribution. If an annotation was not possible based on these parameters, then the metabolite exact mass (neutral) is given. The evidence collated for each metabolite discussed in this manuscript is summarised in S1 Table.

Classification model—Classification models based on Bayesian logistic regression [35] were built in order to provide a system to distinguish stage 1 from advanced stage 2 and to distinguish control from infected subjects in plasma. Each individual LC-MS peak was placed into its own logistic regression model predicting disease state and the deviance calculated. The fifty peaks with the lowest deviance were then picked for further analysis as follows. A recursive feature elimination algorithm [36] was run 10 times (Monte-carlo cross validation) to select the best predictors of disease stage, using a maximum of 2 predictors with a logistic regression model. At each run, 10 sub-runs (Monte-carlo cross validation) each calculated the area under the receiver operating characteristic curve (AUROC) as the metric to maximise. The results of the feature elimination algorithm were an ordered list of the best predictors. Due to the amount of data (20 samples in each condition), it was decided to develop a model with a maximum of two predictors. The top predictor was found to be m/z 216, which had a strong and well-separated LC-MS signal. In examining the next predictor, m/z 133 was found to increase performance, have a strong and well-separated LC-MS signal and be identifiable as ornithine, and was therefore chosen as the second factor. Model performance was calculated using 1000 repetitions of Monte-carlo cross validation (AUROC = 92%, Sensitivity = 92%, Specificity = 81%). This was achieved by taking re-sampled subsets of the data and calculating AUROC and then calculating the mean and standard deviation over the subsets. The given sensitivity and specificity were chosen to be at the operating point closest to the perfect sensitivity and specificity. In developing the model to distinguish control from infected subjects, the same procedure as above was followed. In addition, a model using the same predictors as the first model was developed. There was no significant difference between the two procedures, therefore the model using the same predictors as the first model was shown.

## Results

Twenty stage 1 patients, 20 stage 2 and 16 controls (serological suspects in whom parasites were not found) were randomly selected from a larger cohort and used for metabolomics analysis. Characteristics of these subjects are summarised in Table 1.

**Table 1:** Characteristics of the studied subjects

	Stage 1	Stage 2	Controls
Number of patients	20	20	16
Sex (male/female)	10/10	13/7	6/10
Age (median, range)	43 (17–83)	35.5 (12–62)	35 (12–70)
CATT positive (positive/number tested)	20/20	20/20	16/16
(CATT titre range)	(8–32)	(16–32)	(16–32)
Presence of trypanosomes* (lymph, blood, CSF*)	(4,16,0)	(9,10,20)	(0,0,0)
CSF WBC** per μL (median, range)	1 (0–4)	200 (50->1000)	3 (1–250)
Symptoms at general examination (by medical doctor)***	13/20	19/20	6/16
Sleep disturbances****	6/20	20/20	3/16
Neurological signs*****	7/20	20/20	5/16

*Trypanosomes seen in examined biofluid. CSF: cerebrospinal fluid;

** WBC = white blood cells;

***Summarises the clinical impression of a medical doctor after patients’ general physical examination (before neurological and psychiatric assessment).

****Measured using specific items from the MINI test [28] and Hamilton scale [37].

*****Presence of at least one neurological sign suggestive of HAT, including sleep and psychiatric disturbance.

Advanced stage 2 patients were clearly identified clinically with the presence of neurological signs and CSF cell counts above 20 WBC/μL. Although a lumbar puncture was performed to confirm diagnosis, a trained practitioner could have made the diagnoses clinically in all 20 cases. Stage 1 patients are not easy to ascertain clinically. Here, 13/20 presented with symptoms, but only seven with signs more specific to HAT. Signs and symptoms from these patients are presented in S2 Table. Control patients presented general symptoms in six cases as well as having tested CATT positive indicating the possible presence of HAT or other diseases.

All included patients were negative for HIV and syphilis. One stage 1 patient was identified microscopically with malaria at diagnosis. None of these patients showed evidence of other co-infections.

The control group comprised individuals who were serologically positive using the CATT test at high titres (16 or 32), but in whom parasites were not found using microscopy combined with concentration techniques. It is not ethically acceptable to extract CSF from patients in whom the disease is not initially suspected, so seronegative controls from the same region could not be sought. The CATT test is not 100% specific and will produce some false positives, however, it is also possible that some patients in our control group did contain undetected trypanosomes. Among the control subjects, one presented microfilaria in the blood, one had signs of bronchitis and one had rheumatoid pains. Given the high level of clinical suspicion and presence of raised CSF cells, three subjects were treated with eflornithine. Nine control patients were followed without any treatment and five became seronegative during follow up while the remaining four were lost to follow up, and their outcome was unknown. The five seropositive patients that became seronegative either “self-cured” or were never infected.

Spectral profiles—After data processing and filtering, 1,021 robust metabolite features (features that were reproducible in terms of mass and retention time, above an intensity cut-off and present in at least three samples of a sample group) were detected in patient plasma, 512 in CSF and 694 in urine. The high number for plasma reflects the richness of this biofluid compared to CSF. The lower number in urine compared to plasma is likely due to computational filtering based on the concentrations of the metabolites being so variable that the software was unable to include in meaningful analysis. Total ion count variability between samples within groups was below 0.06 relative standard deviation in CSF and plasma samples, and the number of metabolite features detected for each patient in a group did not vary significantly in plasma or CSF (S1 Fig).

**Fig 2:**
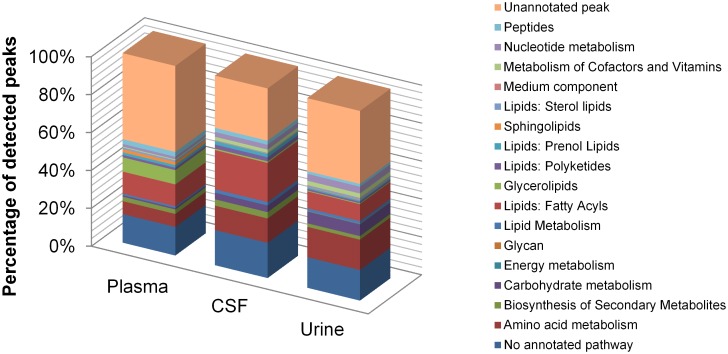
Variation in metabolic coverage of each biofluid. The percentage shows the number of metabolites detected in each metabolic class (based on matches to the IDEOM database) as a percentage of the total number of metabolites detected in all three groups. Unannotated peaks: masses with no IDEOM database match, no annotated pathway: metabolites that did not match databases for known metabolic pathways. Medium components are those that are commonly found in trypanosome growth medium.

Around half of the plasma peaks could not be annotated for a metabolite identity, which is usual for untargeted metabolomics data, given the large numbers of metabolites that have yet to be formally characterised chemically [38]. A larger proportion of the metabolites in CSF and urine were putatively annotated. The coverage of the metabolome varied somewhat between the three different biofluids, with more fatty acyls detected in CSF compared to urine or plasma, and more glycerolipids discovered in plasma (Fig 2). We specifically sought the trypanosomatid specific metabolite trypanothione, but could not identify it in samples, probably because the low parasitaemia characteristic of *T*. *b*. *gambiense* infections would assure it remains below the detection limit (5 nM). It has been shown that trypanosomes also secrete large quantities of keto acid derivatives of the aromatic amino acids, e.g. phenylpyruvate, enol-phenylpyruvate, indole pyruvate and indole lactate [39], however, these were not detected in blood or CSF, which again is likely to be attributable to the low parasitaemia.

**Fig 3:**
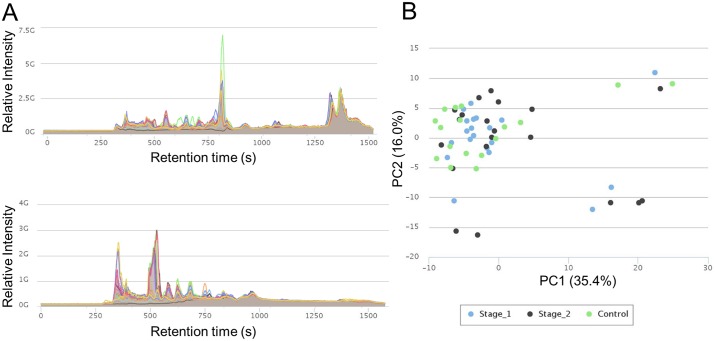
Variability across urine samples. (A) Total ion chromatograms (TICs) show the range in concentration of the ions between samples. Ions are analysed in positive (top) and negative (bottom) ionisation modes. The control group is shown as an example. (B) Principal components analysis shows a lack of separation of the sample groups in raw data.

Urine metabolites vary in concentration making biomarker discovery challenging—An analysis of the total ion chromatogram for each urine sample revealed a very large variation in the concentration of the samples (Fig 3a). This is a common problem in urine samples as the water levels in the samples are not controlled. There are debates on the best method to normalise urine sample data to account for the wide variation in concentration [40]. Creatinine levels are often used for this type of normalisation, but can cause over fitting of the data, resulting in amplification of irrelevant differences [33]. A principal components analysis of the urine data showed that the samples did not cluster into groups, either before or after creatinine normalisation (Fig 3b and S2 Fig). Univariate analysis of individual metabolites also failed to identify any whose abundance clearly varied between patients at stage 1 or advanced stage 2 and controls.

**Fig 4:**
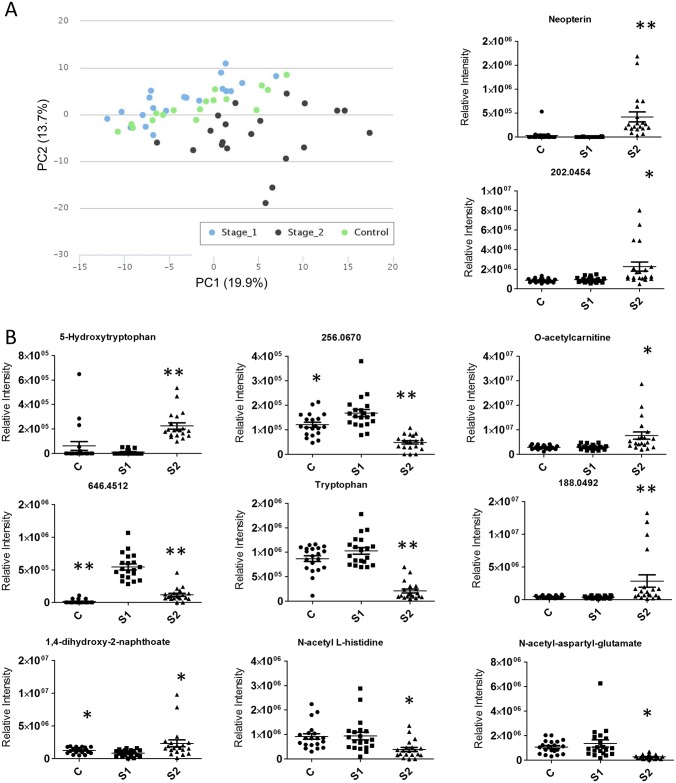
Separation between the metabolite patterns of stage 1 and advanced stage 2 CSF samples. (A) Principal components analysis plot. (B) Histograms for metabolites showing significant differences between control (C), stage 1 (S1) and advanced stage 2 (S2) infected patients. * indicates p<0.05 compared to stage 1, ** indicates p<0.001 compared to stage 1 in a Students t-test. Bars show standard error of the mean. Relative intensities measure peak areas.

CSF findings—HAT and control patients separated slightly in a principal components analysis plot (Fig 4a). Previous studies have used neopterin in CSF as a marker for stage 2 trypanosome infection; our data confirmed neopterin as a good marker with a sensitivity and specificity of 100% for discrimination between stage 1 and advanced stage 2 infections (Fig 4b). This finding also corroborates the untargeted metabolomics approach as offering unrivalled potential for novel biomarker discovery for HAT. Four control patients (A010C, A011C, A013C and A020C) showed high levels of neopterin in their CSF above the cut-off in the ROC (receiver operating characteristic) curve of 24,342 (S3 Table).

**Fig 5:**
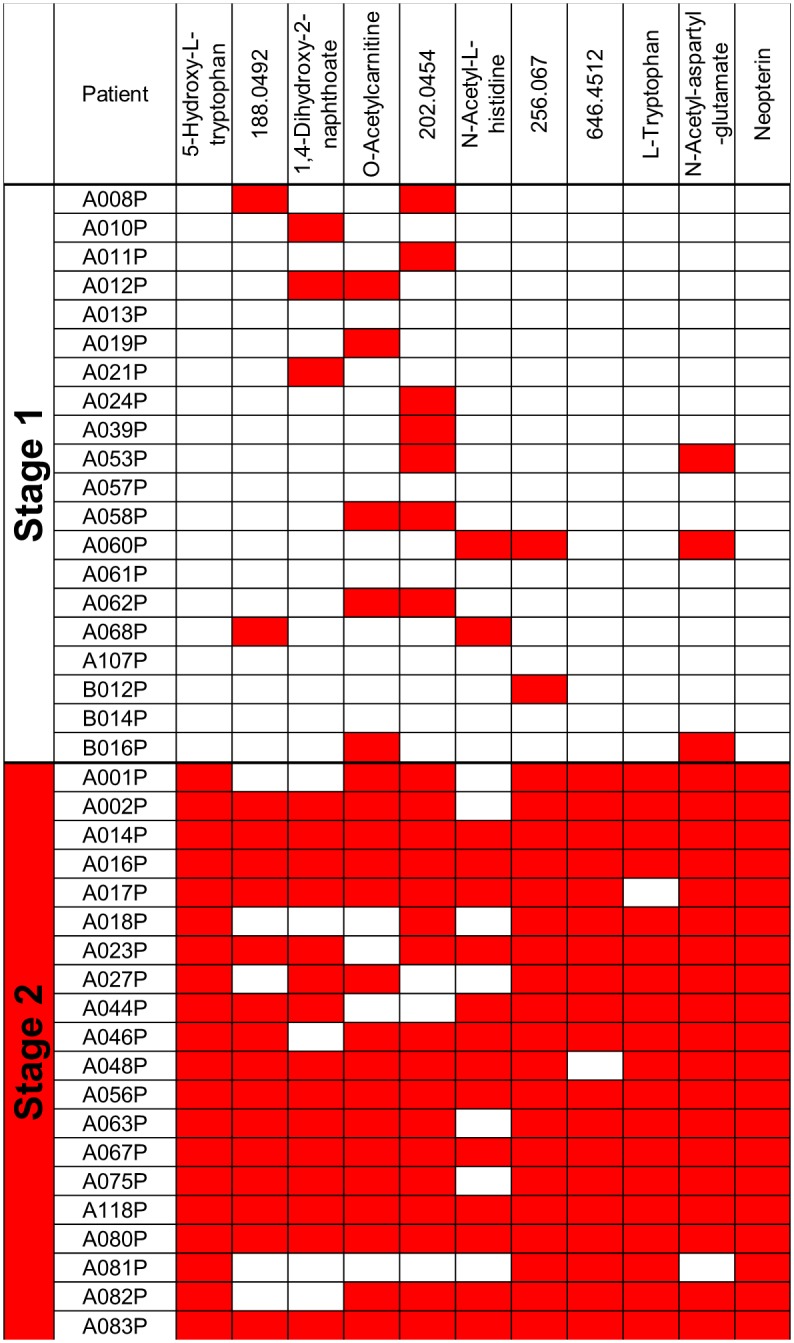
Patients can be classified into stage 1 and advanced stage 2 groups using eleven biomarkers in CSF. O-acetylcarnitine and tryptophan match to authentic standards. Some masses did not match to metabolites in the IDEOM [32] database and are identified by the mass only. Red shading indicates peak area intensities above/below the cut-off for advanced stage 2 disease. More information on the cut-offs are shown in S4 Table.

Several metabolites were altered following trypanosome invasion of the CNS, including an increase in 5-hydroxytryptophan, and a decrease in tryptophan in advanced stage 2 patients. This change was accompanied by a small increase in kynurenine in advanced stage 2 disease (S4 Table). The ten metabolites (apart from neopterin) showing the greatest significant change in abundance in advanced stage 2 infection compared to stage 1 infection are shown in Fig 4b. Notably, there are several patients with much higher levels of m/z 188, O-acetylcarnitine and m/z 202. Using a cut-off for each of these 10 metabolites, determined by the greatest combined sensitivity and specificity (Gain in certainty [41]) for each metabolite intensity, the individual performance of each biomarker for every patient was determined (Fig 5 and S3 Table).

It is common in untargeted metabolomics experiments to detect numerous metabolites which are not yet represented in databases, given the huge diversity of metabolite space [42]. Indeed, some of the metabolites measured here could not be identified based on their mass and retention time. Fragmentation patterns and isotope distributions also failed to provide clues to their identities. All of these masses are singly charged and have normal carbon isotope patterns. Mass m/z 646.4512 has a single positive charge, a bimodal peak shape, and a predicted formula of C_34_H_68_O_5_N_2_PS. Additional analytical approaches would be required to provide robust identities of these metabolites.

**Fig 6:**
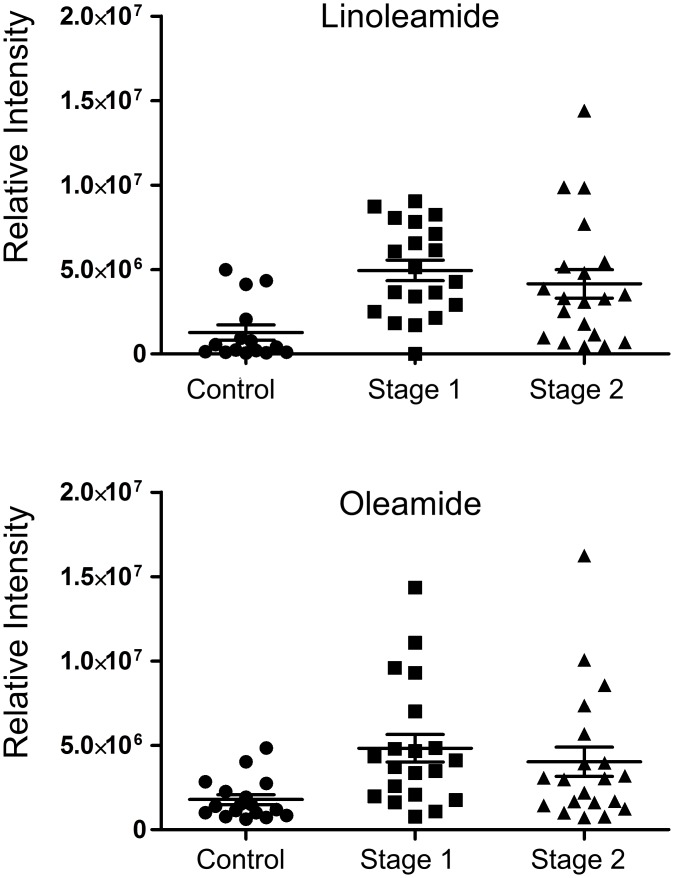
Sleep-inducing metabolites linoleamide and oleamide were increased in both stage 1 and advanced stage 2 patients. Bars show the mean and the standard error of the mean. Metabolite annotations are by mass only. Relative intensities measure peak areas. * indicates p<0.05 compared to control, ** indicates p<0.001 compared to control in a Student t-test.

In addition to 5-hydroxytryptophan, which is a key metabolite associated with somnolence, we also identified metabolites with masses allowing putative annotation (based on mass and formula) as linoleamide and oleamide. These metabolites were both increased in stage 1 and advanced stage 2 samples compared to the control group, albeit without reaching statistical significance (Fig 6). Given the inter-sample variability and low sample size, caution is warranted in interpreting any possible effect. The synthesis of linoleamide has not been studied, but oleamide has been shown to be synthesised from oleic acid and ammonia (and to a lesser extent, glutamine) [43]. The likely precursors of linoleamide and oleamide (linoleic acid and oleic acid) were not detected.

**Fig 7:**
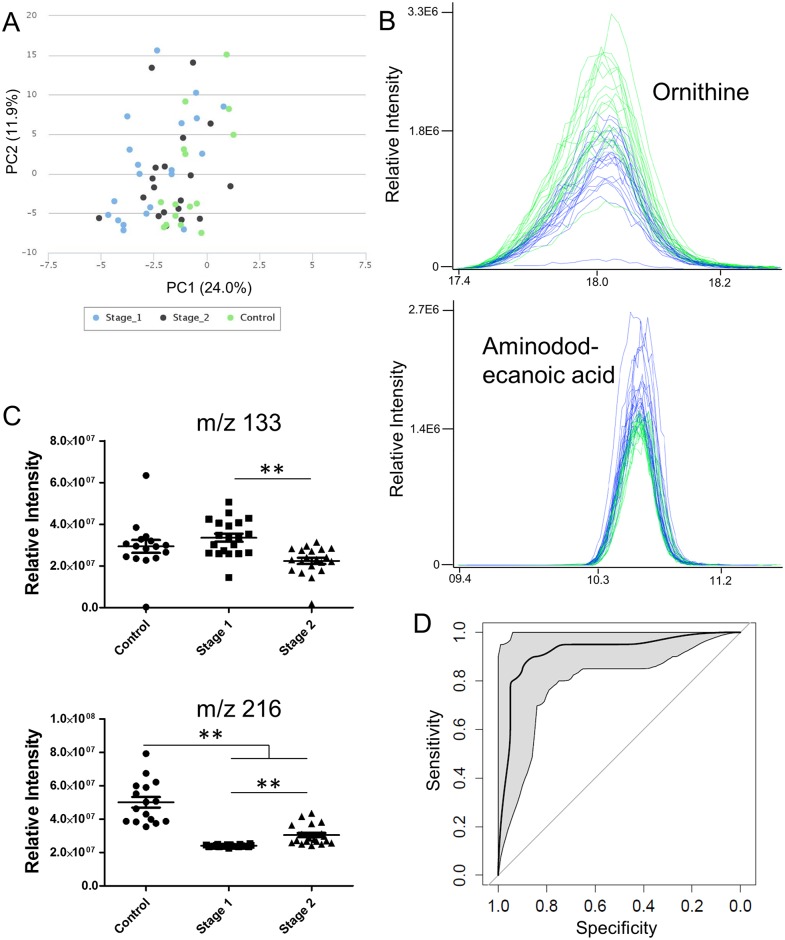
Metabolite differences in plasma are small, but significant. (A) Principal components analysis. (B) Extracted peaks for m/z 133 (ornithine) and m/z 216 (aminododecanoic acid). Stage 1: green, advanced stage 2: blue. (C) Histograms for m/z 133 and m/z 216 (relative intensities measure peak areas). ** indicates a p-value of <0.001 in a Students’ t-test. (D) ROC curve for m/z 133 and m/z 216 showing the 95% confidence intervals.

Biomarkers in plasma—Metabolic features in plasma samples were generally very well regulated, and significant deviations from normal levels were minimal due to normal homeostatic regulation. Sample groups were not separated on a PCA plot (Fig 7a). There were, however, 308 metabolic features whose abundance was significantly altered between stage 1 and control patients, 181 between advanced stage 2 and uninfected, and 237 between stage 1 and advanced stage 2 (S5 Table). None of these significant changes were large, however two masses (Fig 7b and 7c) were able to produce a good model (Fig 7d) that separated stage 1 and advanced stage 2 infections. This model had an area under the ROC curve of 92% and could be marginally improved by adding two more masses (S3 Fig). Confidence intervals for sensitivity and specificity can easily be calculated for any operating point by examining Fig 7d.

M/z 133.0971, detected in positive ionisation, was decreased in advanced stage 2 disease compared to stage 1 and was identified as ornithine (match to standard retention time, fragmentation pattern and expected isotope distribution (S1 Table)). M/z 216.1958, detected in positive ionisation, was increased in advanced stage 2 disease compared to stage 1 and was annotated as aminododecanoic acid (based on its mass plus fragmentation and isotope distribution).

A second model (Fig 7d) was built that separated control and infected subjects using the same peaks as the first model. This model had an area under the ROC curve of 94% and showed 87% sensitivity and 95% specificity at the best point on the curve.

## Discussion

Many studies show metabolite differences that may act as biomarkers of infectious diseases in sub-Saharan Africa [18,44,45], including a recent paper analysing metabolic biomarkers in *T*. *b*. *rhodesiense* infection [25] where changes in amino acid and lipid metabolism compared to uninfected control patients were reported, although robust markers that would be suitable for diagnostics were not proposed [25]. We were able to find changes in the levels of ornithine and aminododecanoic acid in blood that were predictive of both the presence of disease and disease stage. These metabolites were not detected in the study by Lamour *et al*. [25], and so it would be interesting to test samples from *T*. *b*. *rhodesiense* HAT patients to see if they are also altered upon infection in an East African cohort.

A greater challenge for HAT diagnostics, however, is not to diagnose an infection, but to use an alternative to CSF to accurately stage the disease once infection has been detected by microscopy or a serological test. This staging would ideally avoid the need for dangerous lumbar punctures in the field allowing correct treatment to be given.

Urine could be of great value as a source of biomarkers, since its collection is not invasive, and samples can be used with minimal preparation. Several urine based biomarkers have been proposed e.g. for diabetes, prostate cancer [46], bladder cancer [47] and possibly for diseases of the brain [48]. Unfortunately the high degree of variation in urine water content and therefore metabolite concentration confounds its utility. This could be ameliorated by providing controlled volumes of water at predetermined times before collecting urine, although this was not done in the current study, and may not be feasible in large HAT screening campaigns in remote field settings. Variability in urine metabolite levels in this dataset therefore made it difficult to identify metabolites that would be predictive of disease.

CSF is mainly produced by ependymal cells of the choroid plexus in the brain, turning over around four times per day, washing the central nervous system of metabolic waste [49]. CSF contains much less protein than blood plasma. Moreover, pH and levels of different neurotransmitters and various metabolites must be tightly regulated to avoid damage to the brain. In our analysis there were clear differences between the CSF of patients in stage 1 and late stage 2 HAT. Neopterin has previously been seen in stage 2 HAT patients, but its use as a biomarker is limited by the wide range of inflammatory disorders it is predictive of [18,50,51]. That, and the fact that our cohort contained only advanced stage 2 samples, is why specificity is much lower in the field [50]. Four control patients had levels of neopterin over our cut-off. It is possible that these individuals had a separate infection that was not diagnosed, emphasising the limitations of neopterin as a biomarker when used in isolation.

The increase in 5-hydroxytryptophan and the decrease in tryptophan seen in our CSF analysis could be due to an increase in the activity of tryptophan 5-monooxygenase. The depletion in tryptophan levels has been noted before in blood of rodents infected with trypanosomes [52,53] and dietary tryptophan is rapidly metabolised to tryptophol and indole acetic acid [54]. Tryptophan depletion is also common in inflammation due to its conversion into metabolites of the kynurenine pathway (kynurenine also being increased in advanced stage 2 disease) [52,55]. However, we failed to identify significant changes in metabolites of this pathway in blood, and the majority of these metabolites were not detectable in human CSF (5-hydroxytryptophan, kynurenine and tryptophan being the exceptions). Previous studies have shown that single nucleotide polymorphisms in tryptophan 5-monooxygenase could be linked to neuropsychiatric disorders [56]. A substantial increase in 5-hydroxytryptophan is seen in aromatic L-amino acid decarboxylase deficiency, where patients are rendered in a vegetative state [57] and treatments with 5-hydroxytryptophan have been used to treat depression caused by serotonin deficiencies [58]. 5-hydroxytryptophan may be a possible marker of stage 2 infection, and could increase the specificity of neopterin for staging using CSF. It would be interesting to investigate whether 5-hydroxytryptophan levels revert to normal after treatment and could therefore be used as a test of cure.

The increase in levels of putative linoleamide and oleamide in both stage 1 and stage 2 HAT is interesting. These molecules are known to increase in the brains of sleep deprived animals [59–61], and oleamide has been shown to induce sleep when injected into rats [59]. It may be that the changes associated with sleep disturbances in HAT start to occur in stage 1 of the disease, as has previously been suggested [62]. Linoleamide and oleamide may be early markers of sleep disturbance, although further work on the identity of these metabolites (so far based solely on mass) and experiments to determine roles would be needed, as are larger patient cohorts to determine significance.

The CSF metabolites shown in Fig 5 can be used to stage patients with 100% specificity and sensitivity, but will still require a lumbar puncture—something which would be avoided with blood based biomarkers.

Whole blood is routinely taken in the field when screening for HAT by microscopy, and a biomarker that could detect the presence of trypanosomes, while also staging the disease would be ideal. Several biomarkers were found that had significantly altered levels in the blood of advanced stage 2 patients compared to stage 1 patients, and two of these were able to produce a robust model able to stratify patients by disease stage. As shown by the ROC curves in Fig 7d, by using the logistic regression classification models, we were able to find changes in the levels of ornithine and aminododecanoic acid in blood that were predictive of both the presence of disease and disease stage. Ornithine levels were decreased in advanced stage 2 disease compared to stage 1. Ornithine is used by trypanosomes to synthesise polyamines and trypanothione—the main reducing thiol in the cells. In normal adult blood, there is an estimated 50–100 μM of ornithine (www.HMDB.ca) and trypanosomes have been shown to import significant amounts of ornithine [30]. The increased ornithine in the blood of stage 1 HAT patients compared to advanced stage 2 may reflect an increased production in the blood to compensate for the uptake by the parasites. However, the very low parasitaemia in *gambiense* patients would make it doubtful that it is parasite consumption of ornithine that leads to its loss, in which case hitherto unknown roles for ornithine in infection might be at play. The second mass used in the model was putatively annotated as aminododecanoic acid and was increased in advanced stage 2 disease. Aminododecanoic acid is not a naturally occurring metabolite and may be a breakdown product of a nitrogenous lipid.

These metabolites warrant further investigation in another cohort to determine whether they can be used to stage HAT in blood.

Limitations—This pilot study identified several biomarkers, but had a number of limitations.

Firstly, the numbers of patients in the cohort tested were small (16–20 per arm). It would be very interesting to see whether the metabolites identified could be used for diagnosis and staging in a second, larger cohort. If these markers are able to diagnose and/or stage disease in a second cohort, then their development into prototype tests should be prioritised.

Secondly, there is ambiguity as to what constitutes early stage 2 and late stage 1 infection. For this reason, advanced stage 2 patients were compared to stage 1 patients in our study. Analysing the levels of the proposed biomarkers in early stage 2 infections will be vital to validating their use in the field.

Thirdly, the identity of some of the biomarkers could not be achieved using our mass spectrometry platform. To understand the mechanisms behind the changes seen, it would be useful to get true identifications of all the metabolites seen. This could be achieved using stepped fragmentation mass spectrometry or, if levels in the tested biofluid are high enough, nuclear magnetic resonance.

Conclusions—The work reported here reveals the extraordinary power of the untargeted metabolomics approach to identify biomarkers of disease. The finding of metabolites whose abundance is predictive of infection and able to discriminate between stage 1 and advanced stage 2 disease, even using levels of metabolites found in blood, offers the potential of removing the need for lumbar puncture from HAT staging algorithms. These results would, however need to be confirmed using a large, independent cohort of patients, before they can be developed into a useful test.

A rapid diagnostic test developed for HAT screening will need to be cheap, fast and easy to use, used with minimal sample preparation, non-refrigerated and accurate. The sensitivity and specificity of the test will depend on whether active or passive screening is required. For active screening, a more sensitive test may be required to detect more cases, whereas in passive screening more specificity may be required to avoid false positives. The levels of the metabolites used as a cut-off in our model can therefore be altered to achieve a more desirable sensitivity vs specificity trade-off.

## Supporting Information

S1 Fig The number of features detected for each patient in positive and negative ionisiation after processing through mzMatch [24]. (TIF)

S2 Fig Principal components analysis of urine samples after normalisation to creatinine using Metaboanalyst [33]. (TIF)

S3 Fig Peaks that marginally improve the model for plasma sample classification into stage 1 and advanced stage 2 HAT. (TIF)

S1 Table Data contributing to the identification or annotation of each metabolite discussed in the manuscript. The level of identification attained (according to the Metabolomics Standards Initiative [63]) is shown.
(XLSX)

S2 Table Clinical characteristics of patients included in this study. (XLSX)

S3 Table Patients can be classified into stage 1 and advanced stage 2 groups using eleven biomarkers in CSF. O-acetylcarnitine and tryptophan match to authentic standards. Some masses did not match to a known metabolite in the IDEOM database and are identified by the mass only. Numbers represent relative peak area intensities on a QExactive (Thermo Scientific). Red shading indicates peak area intensities above the cut-off for advanced stage 2 disease. Blue shading indicates peak area intensities below the cut-off for advanced stage 2 disease.
(XLSX)

S4 Table IDEOM [32] file containing metabolomics features associated with each HAT disease stage in CSF samples. The metabolite identities in this table are putative and should not be taken as true identities without further confirmation.
(7Z) Click here for additional data file.

S5 Table IDEOM [32] file containing metabolomics features associated with each HAT disease stage in plasma samples. The metabolite identities in this table are putative and should not be taken as true identities without further confirmation.
(7Z) Click here for additional data file.
