# Supplementary figures and images for: Metabolomics Identifies Multiple Candidate Biomarkers to Diagnose and Stage Human African Trypanosomiasis
Source: PLoS Negl Trop Dis. 2016 Dec 12;10(12):e0005140. doi: 10.1371/journal.pntd.0005140 (PMC5152828; doi:10.1371/journal.pntd.0005140)

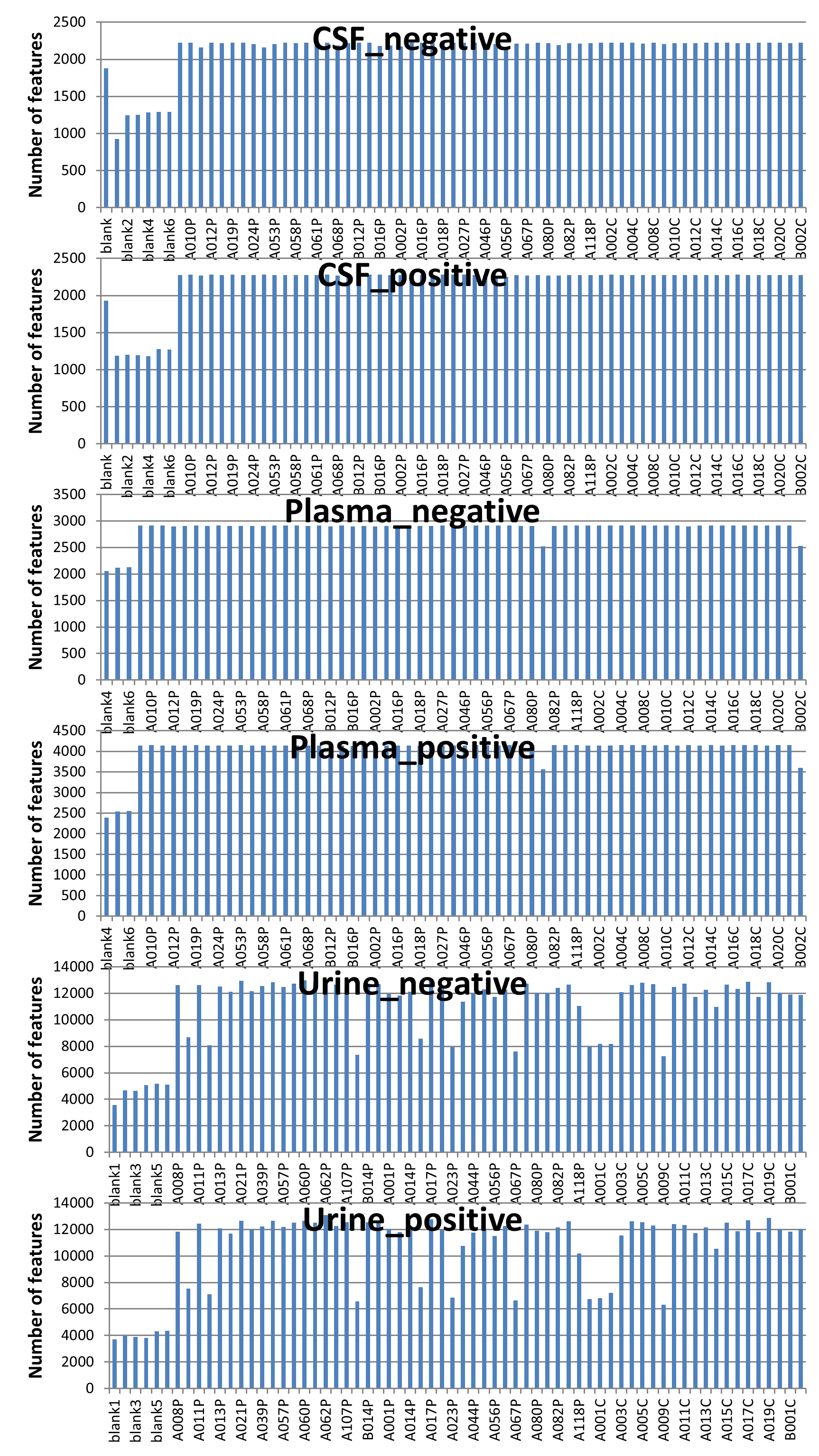

Supplement: S1 Fig — (TIF) [file pntd.0005140.s001.tif]

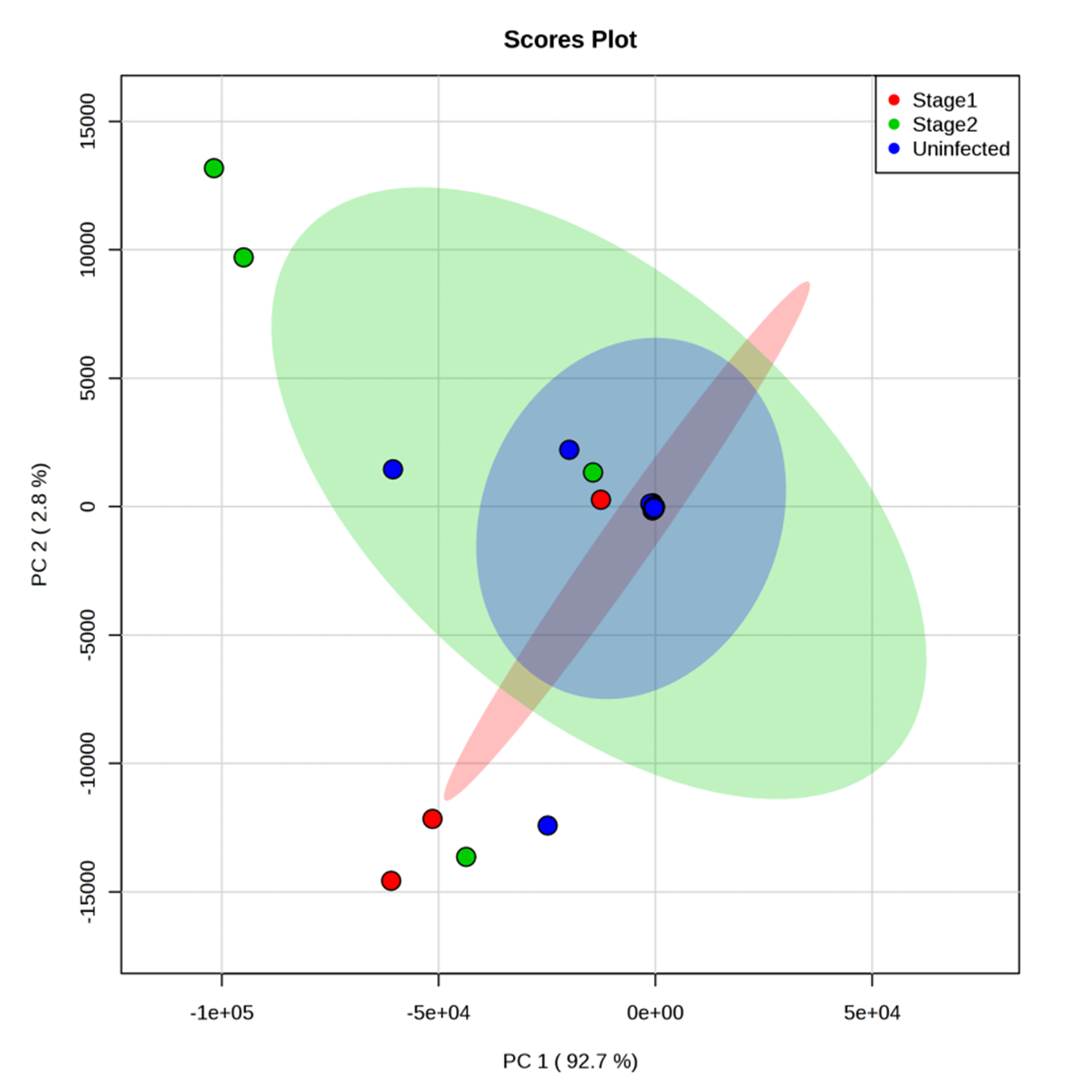

Supplement: S2 Fig — (TIF) [file pntd.0005140.s002.tif]

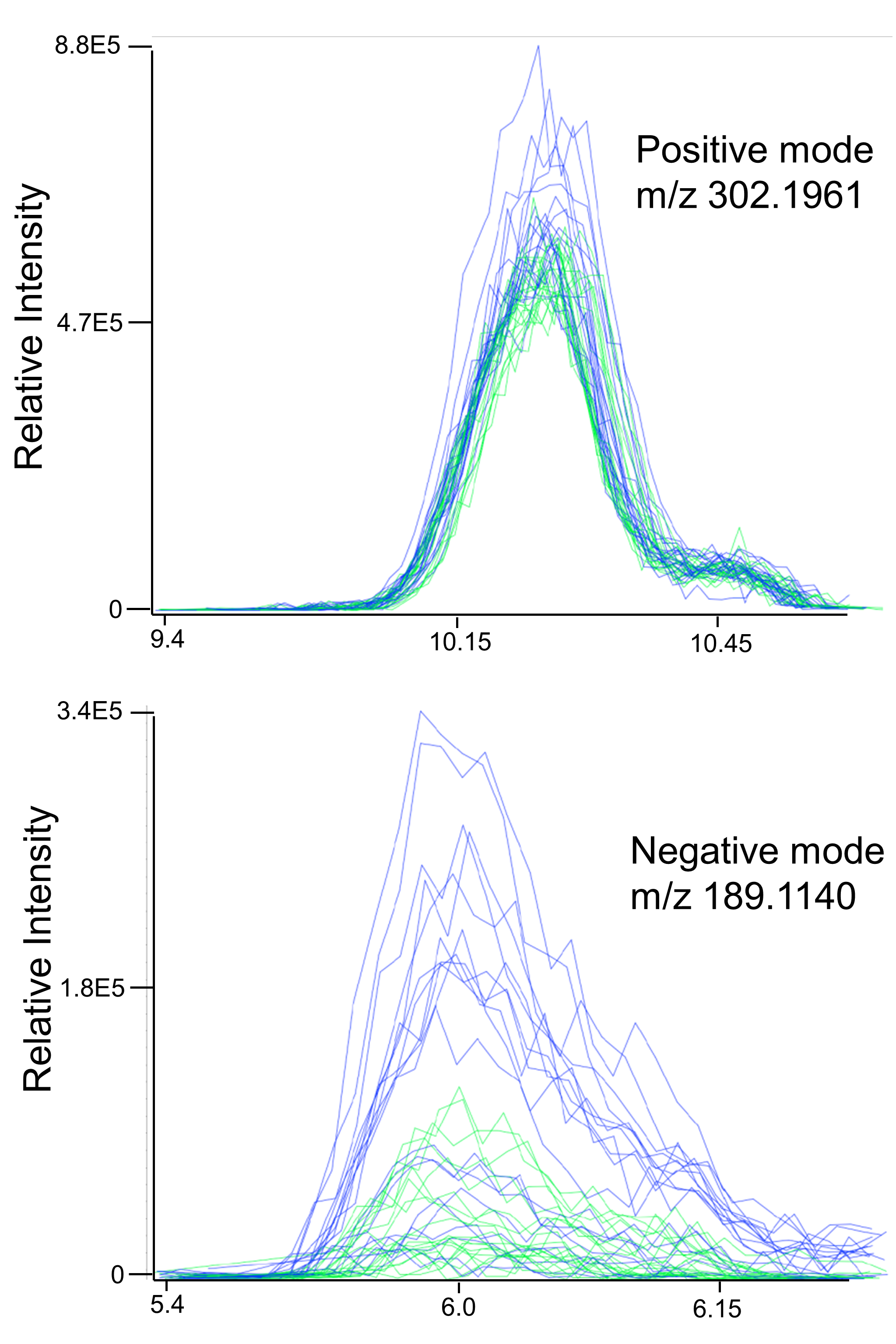

Supplement: S3 Fig — (TIF) [file pntd.0005140.s003.tif]
